# Curing glioblastoma: oncolytic HSV-IL12 and checkpoint blockade

**DOI:** 10.18632/oncoscience.359

**Published:** 2017-09-20

**Authors:** Dipongkor Saha, Robert L. Martuza, Samuel D. Rabkin

**Affiliations:** Molecular Neurosurgery Laboratory and the Brain Tumor Research Center, Department of Neurosurgery, Massachusetts General Hospital and Harvard Medical School, Boston, MA, USA

**Keywords:** oncolytic virus, herpes simplex virus, cancer stem cells, immunotherapy, immune checkpoint

Cancer immunotherapy has recently revolutionized how we approach the treatment of cancers, including fatal glioblastoma (GBM), but still fails to effectively treat the majority of cancer patients [1]. There are several key factors that may contribute to therapy failure, including, but not limited to: (i) low mutational loads and poor tumor immunogenicity; (ii) immune suppressive tumor microenvironment (regulatory T cells, pro-tumoral M2-like macrophages); and (iii) tumor heterogeneity, including therapy-resistant GBM stem cells (GSCs). GSCs contribute to tumor initiation, progression, maintenance, and recurrence, and are thus critical targets for therapy. Recently, we described a new stringent difficult-to-treat stem cell-based immune competent GBM model (005 GSC) that addresses aforementioned features of therapeutic hindrance: low mutational load with only two known somatic mutations; relatively non-immunogenic, lacking MHCI and II expression, with PD-L1 only expressed on a minority of 005 GSCs; highly tumorigenic and invasive; and an immune suppressive tumor microenvironment [2, 3]. This model has been used to test immunotherapeutic strategies for GBM.

**Figure 1:**
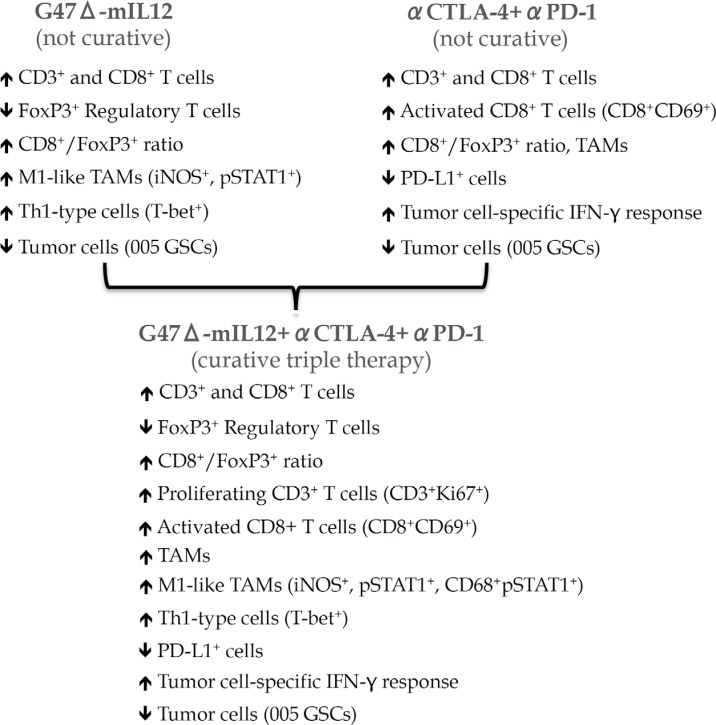
Treatment alterations in tumor microenvironment

Oncolytic viruses [4], e.g. oncolytic herpes simplex virus (oHSV), are a distinct class of anticancer agent with unique mechanisms of action: selective targeting and killing of cancer cells irrespective of tumor mutational load, immune status, and heterogeneity, while sparing normal cells, and exposing viral/tumor antigens, which promote cascades of anti-tumor (and anti-viral) immune responses *(in situ* vaccine) [5]. Despite these properties, oHSV G47Δ, which is currently in clinical trial in Japan, was insufficient alone in the 005 GSC model. However, viral expression of IL12 (G47Δ-mIL12) produced a significant but modest improvement in survival of 005 tumor-bearing mice [2, 3]. This was associated with an increased number of tumor infiltrating T cells, increased effector/regulatory T cell ratio, skewing the tumor-associated macrophages (TAMs) towards an anti-tumoral M1-phenotype, and decreased tumor cells (Figure 1) [3].

Immune checkpoints, such as cytotoxic T lymphocyte antigen 4 (CTLA-4) and programmed death 1 (PD-1) play a critical role in regulating immune responses and suppressing immune effector cells. PD-1 and CTLA-4 are expressed predominantly on T-cells, while PD-L1 is also expressed on endothelial and tumor cells. Blocking antibodies to these molecules are effective at reversing tumor-induced immunosuppression [1]. Therefore, we hypothesized that G47Δ-mIL12, which induces antitumor immune responses, should synergize with immune checkpoint inhibitor (ICI) antibodies in impeding GBM growth. In the 005 model, ICI antibody alone (anti-PD-1 or anti-CTLA-4) produced only modest improvement in survival, similar to virotherapy (G47Δ-mIL12) alone, while the combination of G47Δ-mIL12 with either antibody or combination of two antibodies further extended survival modestly [3]. The limited efficacy of the dual combination was not due to the inability of antibodies to cross the blood brain/tumor barrier, since antibodies were detected in the tumor. However, the triple combination of G47 Δ-mIL12+anti-CTLA-4+anti-PD-1 cured 89% mice with 005 tumors and protected them from lethal tumor re-challenge. These findings were reproduced in another aggressive immune competent glioma model, CT-2A [3]. Though single or dual therapies significantly modulated the tumor microenvironment, triple combination therapy produced the most prominent anti-tumor effects, such as a significant reduction in tumor cells, influx of M1-like TAMs, increased proliferating T cells, and an increased T effector/regulatory cell ratio (Figure 1). Depletion studies demonstrated a requirement for CD4+, CD8+ T cells, and macrophages for therapeutic efficacy, with CD4+ T cells playing an essential role [3]. It remains to be determined which factor/gene(s) were responsible for the complex immune cell interactions and how they contributed to CD4+ T cell-dependent therapeutic benefit. Whether triple therapy using other oncolytic viruses results in similar curative benefits will be important to determine.

An oHSV encoding human granulocyte-macrophage colony-stimulating factor (GM-CSF) (Talimogene laherparepvec or T-VEC), similar to G47Δ-mIL12, was recently approved for the treatment of patients with advanced melanoma, an immunogenic tumor [6]. Follow-on clinical trials with T-VEC in combination with anti-CTLA-4 (ipilimumab) in melanoma elicited significant clinical responses, with a durable response rate of 50% [7]. However, triple combination therapy (oHSV+anti-CTLA-4+anti-PD-1) may be required to obtain curative responses in the majority of cancer patients with non-immunogenic or ICI non-responding tumors, as described for GBM [3].

An important issue to be addressed before the full potential of oHSV-based cancer immunotherapy is realized is maximizing oHSV replication/spread within the tumor and developing representative preclinical models. For example, oHSV replication is limited in mouse tumors [2], and anti-viral immune responses can limit virus spread in patients. Therefore, developing strategies to enhance tumor-specific viral replication and spread, and anti-tumor immunity without compromising safety is key for clinical success. More research is needed to optimize new viral vectors and design more rationale combination clinical trials. This may include the generation of new oHSV vectors expressing other immune modulators, testing them in combination with other immunotherapies, and expanding clinical development to patients with minimally immunotherapy responsive lethal cancers, like GBM.
